# Neuroinflammation and Secretase Regulation in Alzheimer’s Disease: From Molecular Cross-Talk to Multi-Target Therapeutics

**DOI:** 10.3390/ijms27114824

**Published:** 2026-05-27

**Authors:** Giovanni Luca Cipriano, Ivan Anchesi, Ivana Raffaele, Maria Francesca Astorino, Aurelio Minuti, Marco Calabrò, Concetta Crisafulli

**Affiliations:** 1 IRCCS Centro Neurolesi “Bonino-Pulejo”, Via Provinciale Palermo, Contrada Casazza, 98124 Messina, Italy; giovanniluca.cipriano@irccsme.it (G.L.C.); ivan.anchesi@irccsme.it (I.A.); ivana.raffaele@irccsme.it (I.R.); 2 Department of Biomedical and Dental Sciences and Morpho-Functional Imaging—BIOMORF, University of Messina, 98125 Messina, Italy; mastorino@unime.it (M.F.A.); mcalabro@unime.it (M.C.); ccrisafulli@unime.it (C.C.)

**Keywords:** Alzheimer’s disease, neuroinflammation, secretases, amyloidogenesis, BACE1, ADAM10, γ-secretase, δ-secretase, microRNA, synaptic dysfunction

## Abstract

Alzheimer’s disease (AD) is a progressive neurodegenerative disorder characterized by amyloid-β (Aβ) plaque deposition, neurofibrillary tau tangles, synaptic dysfunction, and progressive cognitive decline. AD is increasingly recognized as a condition in which chronic neuroinflammation actively shifts amyloid precursor protein (APP) processing toward the amyloidogenic pathway, driving Aβ production and accumulation rather than merely accompanying amyloid deposition. In this review, we examine the molecular cross-talk between inflammatory signalling and secretase regulation, highlighting how pro-inflammatory mediators promote amyloidogenic processing and contribute to downstream synaptic dysfunction. We discuss the major pathways linking glial activation to aberrant APP cleavage, including STAT3-dependent BACE1 upregulation, immune-mediated modulation of γ-secretase through IFITM3, and activation of the C/EBPβ/δ-secretase axis, which connects inflammatory stress to both amyloid and tau pathology. We further address the contribution of epigenetic mechanisms, particularly microRNA-mediated derepression of BACE1 and suppression of ADAM10, as well as SIRT3-related impairment of Aβ clearance. These interconnected processes establish a feed-forward pathogenic network in which neuroinflammation amplifies secretase imbalance, amyloidogenesis, and synaptic vulnerability. Finally, we discuss emerging multi-target therapeutic strategies aimed at modulating inflammatory signalling, restoring non-amyloidogenic APP processing, and preserving proteostatic and synaptic resilience. Collectively, this framework supports the view that targeting the inflammatory control of secretase activity may represent a biologically relevant strategy for disease modification in AD.

## 1. Introduction: Neuroinflammation, Secretase Dysregulation, and Synaptic Vulnerability in Alzheimer’s Disease

Alzheimer’s disease (AD) is a progressive neurodegenerative disorder characterized by worsening cognitive impairment, including memory decline, reduced reasoning abilities, and behavioral disturbances, ultimately leading to loss of functional independence [1]. Its global prevalence continues to rise in parallel with population ageing, with dementia currently affecting nearly 50 million individuals worldwide and projected to increase markedly in the coming decades [2]. Neuropathologically, AD is defined by the accumulation of amyloid-beta (Aβ) plaques and neurofibrillary tangles in brain regions subserving cognition [3].

Although Aβ deposition and tau pathology remain the defining histopathological hallmarks of AD, increasing evidence supports a more complex pathological landscape in which aberrant protein accumulation, impaired proteostasis, and chronic glial activation converge to promote amyloidogenic processing and progressive neuronal injury [4,5,6].

Notably, within this framework, early functional alterations at the synaptic level have been observed and may contribute to cognitive decline, although they are likely the consequence of a broader cascade of events rather than a primary pathogenic driver of AD [4,5].

While transgenic models have been instrumental in clarifying familial AD mechanisms, the pathogenesis of sporadic AD remains incompletely understood and is thought to involve a multifactorial interplay among genetic susceptibility, vascular and metabolic comorbidities, and environmental stressors [7].

In this context, increasing attention has focused on the reciprocal interaction between neuroinflammation and secretase dysregulation as a mechanistic axis linking extracellular inflammatory cues to amyloidogenic APP processing and, ultimately, to synaptic vulnerability. This review therefore examines how neuroinflammatory signalling regulates secretase activity and APP processing in AD, with particular emphasis on the molecular pathways that sustain amyloidogenesis and propagate towards synaptic dysfunction.

### 1.1. The Bidirectional Cross-Talk: The Pro-Inflammatory Cytokine Background

The pathogenesis of AD is strictly correlated to a bidirectional cross-talk between Aβ accumulation and a chronic pro-inflammatory cytokine environment [8]. The deposition of Aβ actively stimulates local microglia and astrocytes, enhancing the continuous secretion of pro-inflammatory cytokines such as interleukin-1β (IL-1β), interleukin-6 (IL-6), and tumor necrosis factor-alpha (TNF-α) [9]. Exposure to these Aβ aggregates results in chronic microglial activation, which directly causes the generation of reactive oxygen species and neurotoxic inflammatory mediators [9].

Neuroinflammation in AD should not be regarded solely as a secondary response to amyloid deposition. Instead, inflammatory signalling actively reshapes APP processing and contributes to the maintenance of a feed-forward pathogenic loop in which Aβ accumulation promotes cytokine release, while inflammatory mediators further shift APP metabolism towards the amyloidogenic pathway [8,9,10]. In this framework, cytokine signalling becomes a permissive molecular environment for sustained amyloidogenesis rather than a passive by-product of plaque formation [11].

A major component of this inflammatory transduction is the STAT3 pathway. Systemic inflammatory stimuli, including lipopolysaccharide (LPS)-induced immune activation, promote STAT3 phosphorylation in the hippocampus, where phosphorylated STAT3 translocates to the nucleus and acts as a positive transcriptional regulator of BACE1, increasing BACE1 expression and enzymatic activity. BACE1 is the β-secretase responsible for the rate-limiting step of amyloidogenic APP cleavage [11]. In parallel, persistent STAT3 activation is associated with increased IL-6, IL-1β, and TNF-α signalling, thereby reinforcing chronic glial reactivity and sustaining a neurotoxic inflammatory state [11,12,13].

Defective Aβ clearance may further intensify this inflammatory–secretase axis. In this regard, downregulation of the mitochondrial deacetylase SIRT3 has been associated with reduced expression and activity of key Aβ-degrading enzymes, including insulin-degrading enzyme (IDE) and neprilysin [14,15]. Given the broader role of SIRT3 in metabolic and cellular homeostasis, its deficiency may represent a point of convergence between metabolic dysfunction, impaired proteostasis, and progressive amyloid accumulation in AD [14,16].

### 1.2. Transcriptional Upregulation and Secretase Dysregulation

Aβ is produced through sequential cleavage of APP by secretase enzymes, a heterogeneous group of proteases that operate via different catalytic mechanisms to hydrolyze peptide bonds [17]. When the regulation of these proteolytic enzymes is impaired, APP processing is increasingly directed toward the amyloidogenic pathway. This shift leads to excessive Aβ production and accumulation, which in turn promotes aggregation and exerts neurotoxic effects. Within this pro-inflammatory environment, transcriptional upregulation and functional modulation of distinct secretase enzymes promote activation of the amyloid cascade [18].

Among these enzymes, BACE1, first cloned in 1999 [19,20], represents the rate-limiting β-secretase in amyloidogenic APP processing and is strongly regulated at the transcriptional level by inflammatory signalling. In particular, STAT3 activation has been shown to enhance BACE1 expression under pro-inflammatory conditions, thereby providing a direct mechanistic link between cytokine signalling and increased Aβ production [11,20,21]. Inflammatory cues also modulate γ-secretase function. In response to neuroinflammatory stress, cytokine signalling induces the expression of interferon-induced transmembrane protein 3 (IFITM3) in neurons and astrocytes. IFITM3 physically associates with the γ-secretase complex and enhances its catalytic activity towards APP-derived substrates, thereby increasing Aβ production [10,22,23].

Pro-inflammatory cytokines classically associated with AD neuroinflammation, including IL-1β, IL-6, and TNF-α, can induce expression and nuclear activation of the age-related transcription factor C/EBPβ in glial cells [24]. In this context, Aβ accumulation may promote C/EBPβ activation in part by engaging microglial inflammatory responses rather than acting as an isolated upstream signal, since Aβ and cytokines such as IL-6 can cooperatively enhance C/EBPβ activity and establish a feedback loop between amyloid and neuroinflammation [24,25]. Activated C/EBPβ directly regulates the expression of δ-secretase (asparaginyl endopeptidase/AEP), which cleaves APP and Tau, thereby linking inflammatory signalling to both amyloidogenic and tau-related pathology [26]. Through this C/EBPβ–δ-secretase axis, inflammatory and amyloidogenic signals can form a feed-forward network that progressively amplifies Aβ production, Tau truncation, neuroinflammation, and cognitive decline in AD models [24,26,27,28].

**Figure 1:**
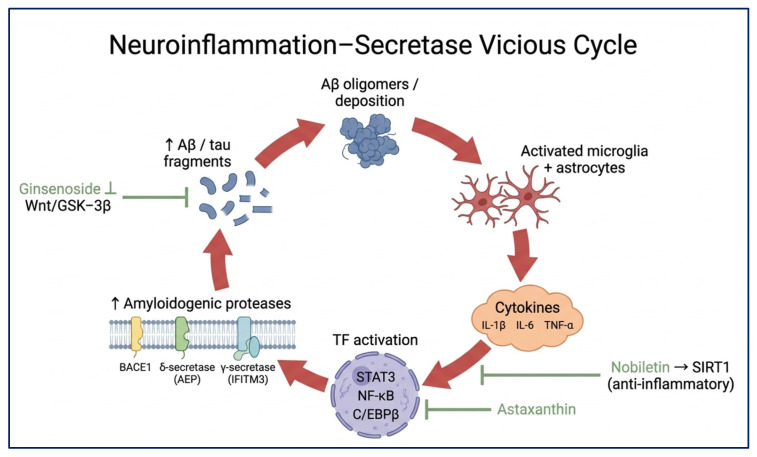
Schematic representation of the neuroinflammation–secretase feedback loop driving amyloidogenesis in Alzheimer’s disease. Aβ deposition activates microglia and astrocytes, promoting the release of pro-inflammatory cytokines, including IL-1β, IL-6, and TNF-α. These mediators activate inflammatory transcription factors such as STAT3, NF-κB, and C/EBPβ, which enhance amyloidogenic secretase pathways, including BACE1, δ-secretase/AEP, and γ-secretase modulation, thereby sustaining further Aβ generation and chronic glial activation.

Beyond direct transcriptional control, the secretase-mediated amyloidogenic pathway may also be regulated by epigenetic factors, likely involving microRNA (miRNA) dysregulation. Indeed, it was observed that pro-inflammatory signalling, particularly via the NF-κB pathway, induces a significant downregulation of miR-338-5p in hippocampal neurons [29]. This miRNA deficiency leads to the transcriptional derepression of BACE1, as miR-338-5p directly targets the 3′-untranslated region (3′-UTR) of the BACE1 mRNA to inhibit its translation [29]. The resultant increase in BACE1 protein levels facilitates the processing of APP into Aβ, which in turn, further exacerbates neuroinflammatory signalling through microglial and receptor-mediated pathways, including NF-κB signalling pathway (Figure 1).

### 1.3. Epigenetic Regulation of Secretases: miRNA Networks and Sirtuin Dysregulation

As previously described, pro-inflammatory signalling triggers an epigenetic layer of control that derepresses amyloidogenic pathways, mainly through downregulation of miR-338-5p in hippocampal neurons. The resulting increase in Aβ oligomers in turn activates NF-κB signalling; primarily through microglial pattern-recognition receptors such as TLR2/TLR4 and RAGE, triggering MyD88/IKK-dependent phosphorylation and degradation of IκBα [30]. This enables NF-κB nuclear translocation and the transcriptional upregulation of pro-inflammatory cytokines, thereby amplifying neuroinflammatory responses. Interestingly, the epigenetic controls may also actively silence protective pathways: neuroinflammation elevates circulating and brain miR-140-3p and miR-122-5p, which act as molecular silencers of ADAM10 α-secretase by targeting its 3′-UTR; overexpression in APP/PS1 mice reduces ADAM10/sAPPα, impairs microglial chemotaxis toward plaques, and exacerbates neurite dystrophy independently of CX3CL1/TREM2 [29,31].

**Figure 2:**
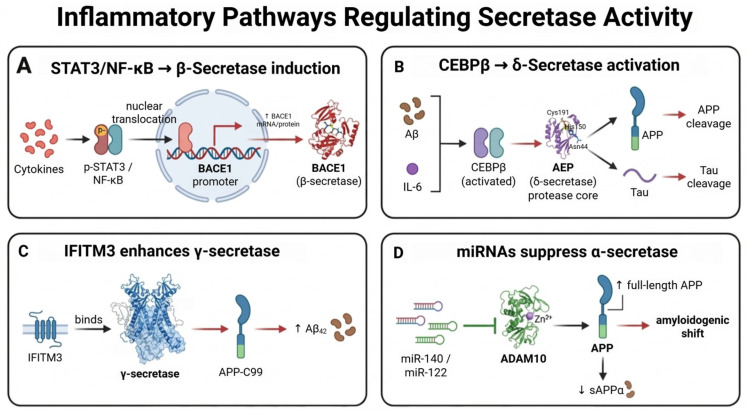
Converging inflammatory pathways regulating secretase activity in AD. (**A**) Pro-inflammatory cytokines (IL-6, TNF-alpha) induce STAT3 phosphorylation and nuclear translocation, driving beta-secretase (red hexagon) transcriptional upregulation via direct promoter binding. (**B**) Amyloid-beta oligomers and IL-6 synergistically activate CEBP-beta in glial cells, promoting delta-secretase (purple triangle) expression that cleaves both APP (N373/N585) and Tau (N255/N368). (**C**) IFITM3, induced by innate immune signalling, physically associates with the gamma-secretase complex (blue circle) to allosterically enhance its activity toward APP-CTF, increasing Amyloid-beta 42 production. (**D**) Neuroinflammation elevates miR-140-3p and miR-122-5p, which post-transcriptionally suppress alpha-secretase (green square) by binding its 3-prime UTR, reducing sAPP-alpha generation and shifting APP processing toward the amyloidogenic pathway. Distinct geometric shapes differentiate secretase classes; red arrows indicate pathological upregulation.

This dual miRNA dysregulation creates selective β-secretase de-repression coupled to α-secretase silencing (Figure 2), explaining heterogeneous hippocampal vulnerability. Furthermore, inflammation-induced mitochondrial deacetylase SIRT3 deficiency, exacerbated by Western diet in Sirt3 ^−/−^;APP/PS1 models, impairs Aβ-degrading enzymes IDE and neprilysin while elevating BACE1; SIRT3 activation via nicotinamide riboside restores IDE/neprilysin and reduces plaques [14]. 

Beyond miRNA-mediated regulation, emerging evidence suggests that long non-coding RNAs (lncRNAs) may provide an additional epigenetic layer linking neuroinflammation to secretase imbalance in AD. Among the most relevant candidates, BACE1-AS appears particularly noteworthy because it is elevated in AD and promotes amyloidogenic signalling by stabilizing BACE1 expression [32,33]. In parallel, lncRNA 51A may favour abnormal APP processing by altering SORL1 splicing and thereby weakening a major sorting pathway that normally limits amyloidogenic APP routing [34,35]. Inflammation-associated lncRNAs such as NEAT1 further support the possibility that non-coding RNA networks integrate microglial activation, BACE1-related signalling, and neuronal vulnerability [34]. Although current evidence remains less mature than that available for miRNAs, these findings suggest that lncRNAs may act as upstream amplifiers of the neuroinflammation–secretase–amyloidogenesis axis and deserve further mechanistic investigation [34].

These epigenetic mechanisms integrate seamlessly into the broader vicious cycle of neuroinflammation and amyloidogenesis. Aβ oligomers activate NF-κB signalling, which reprograms the miRNA landscape by downregulating miR-338-5p while elevating miR-140-3p/miR-122-5p, thereby derepressing BACE1 and silencing ADAM10 [29]. Concurrently, SIRT3 deficiency impairs Aβ clearance via IDE/neprilysin downregulation, creating a perfect storm where amyloidogenic processing accelerates while degradation collapses. This multilayered epigenetic modulation functions as a circuit-specific amplifier, selectively destabilizing vulnerable hippocampal networks and perpetuating secretase imbalance across disease stages [14,29,36].

An integrative overview of these neuroinflammation–secretase regulatory pathways is provided in Figure 2, which the reader may refer to throughout Section 2, Section 3, Section 4 and Section 5.

## 2. Molecular Mechanisms Linking Neuroinflammation to BACE1 Upregulation and Amyloid Processing

Neuroinflammatory activation can be initiated through distinct systemic and central pathways, both of which converge on aberrant APP processing and BACE1 upregulation. Systemic inflammation, commonly modelled by intraperitoneal administration of lipopolysaccharide (LPS), induces peripheral immune activation that signals across the blood–brain barrier and promotes pronounced microglial activation in the hippocampus [37,38]. A complementary model of central metabolic inflammation is represented by intracerebroventricular streptozotocin (STZ), which impairs insulin signalling and induces localized brain insulin resistance [39,40]. In this context, central metabolic stress rapidly increases oxidative stress and augments BACE1 levels, thereby favouring sequential APP cleavage and neuronal Aβ production [41].

As the rate-limiting enzyme in Aβ generation, BACE1 is consistently upregulated in response to multiple pathological stimuli, including oxidative stress [8]. During neuroinflammation, reactive oxygen species promote the release of pro-inflammatory mediators, which in turn enhance BACE1 gene expression and amplify APP processing [42]. In addition to transcriptional control, BACE1 translation is regulated by stress-responsive kinases such as protein kinase R (PKR), which induces phosphorylation of eukaryotic initiation factor 2 alpha (eIF2α) under inflammatory conditions [11,43].

At the transcriptional level, NF-κB and STAT3 represent major signalling hubs linking inflammatory stress to BACE1 induction. Inflammatory stimuli activate IκB kinase (IKK), triggering IκB degradation and NF-κB nuclear translocation, which promotes APP expression and enhances BACE1 transcription [9,44]. In parallel, cytokines such as IL-6 and TNF-α induce STAT3 activation in hippocampal neurons, leading to increased BACE1 mRNA expression [11,13,45]. The relevance of this pathway is supported by studies showing that inhibition of STAT3 attenuates LPS-induced microglial activation and reduces hippocampal BACE1 protein levels [11,13,46,47,48].

Additional metabolic stressors, including hypoxia and glucose dysregulation, further enhance BACE1 activity: the BACE1 promoter contains a hypoxia response element (HRE), rendering it particularly responsive to hypoxic or ischemic conditions [49]. Under hypoxic conditions, the transcription factor hypoxia-inducible factor-1α (HIF-1α) escapes ubiquitin-proteasome degradation and stabilizes within the cell, where it binds to hypoxia response elements (HREs) in the BACE1 promoter, thereby increasing BACE1 expression and promoting β-secretase cleavage of APP [50,51]. Similarly, profound metabolic stress, such as that induced by insulin resistance or chronic hyperglycemia, triggers endoplasmic reticulum stress and thereby amplifies Aβ production [14,16]. Interventions such as intermittent hypoxic treatment can significantly modulate the expression of metabolism-regulated genes in the hippocampus and downregulate BACE1 transcript levels, highlighting the tight coupling between cellular bioenergetics and amyloidogenesis [52].

## 3. α-Secretase (ADAM10): The Inhibition of a Neuroprotective Shield

The non-amyloidogenic processing of APP is primarily mediated by α-secretase, particularly the ADAM10 enzyme. Under physiological conditions, ADAM10 cleaves APP within the Aβ sequence, preventing the formation of neurotoxic amyloidogenic peptides and generating the soluble APP alpha (sAPPα) fragment [53]. However, inflammatory stimuli and associated cellular stress downregulate ADAM10 expression and reduce its catalytic activity, thereby diminishing the release of sAPPα [54]. Consequently, impairment of the ADAM10-mediated pathway represents a key dysregulation of proteostasis, shifting APP processing away from the non-amyloidogenic pathway and toward β-secretase-dependent amyloidogenic cleavage.

A key mechanism underlying ADAM10 suppression involves the aberrant upregulation of specific non-coding RNAs within the neuroinflammatory brain. In particular, miR-140-3p and miR-122-5p are markedly increased in both the systemic circulation and brain parenchyma of AD models, where they function as direct post-transcriptional regulators that inhibit ADAM10 mRNA expression [36]. The overexpression of these microRNAs directly targets complementary sequences within the ADAM10 mRNA, leading to robust suppression of ADAM10 protein translation and a marked reduction in its α-secretase activity [36]. Experimental co-expression of miR-140 and miR-122 in hippocampal neurons results in the pronounced accumulation of full-length APP and a significant decrease in sAPPα levels, contributing to cognitive decline and memory impairment in transgenic models. By acting upstream of APP cleavage, these dysregulated microRNAs function as potent post-transcriptional repressors of the non-amyloidogenic pathway, thereby establishing the miR-140/miR-122-ADAM10 axis as a deleterious regulatory mechanism in AD [55]. More broadly, several miRNAs, including miR-140-5p, miR-30a-5p, miR-138, miR-144/451, miR-221, and miR-32533 (via CREB5) downregulate ADAM10, shifting APP processing away from sAPPα production and toward Aβ generation, and are implicated in AD pathogenesis [56,57,58,59].

In the healthy central nervous system, sAPPα exerts neurotrophic effects, promoting neurite outgrowth, enhancing synaptogenesis, and providing trophic support essential for neuronal survival under adverse conditions [36,60]. In the absence of constitutive sAPPα production, neurons display features of neuritic dystrophy, including reduced dendritic length, complexity, and impaired arborization. Administration of recombinant sAPPα can rescue this impaired neurite outgrowth, indicating that the dendritic structural deficits are directly linked to sAPPα deficiency rather than solely to the accumulation of Aβ [36].

Beyond its direct trophic effects on neurons, the depletion of ADAM10-derived sAPPα severely impairs innate immune function, particularly microglial chemotaxis and phagocytic clearance. In the absence of adequate sAPPα signalling, microglia display dysfunctional reactivity, characterized by reduced migration toward amyloid plaques and diminished encasement of toxic Aβ deposits [36,61]. This restrained microglial chemotaxis correlates with an increase in swollen, dystrophic neurites within the juxta-plaque microenvironment [62]. In vitro studies further demonstrate that the attenuated microglial chemotaxis and deficient Aβ phagocytosis, induced by miR-140 and miR-122 overexpression, can be rescued through the exogenous administration of sAPPα [36]. Collectively, ADAM10 suppression and the consequent loss of sAPPα not only deprive neurons of essential survival signals but also impair microglial immunosurveillance, rendering the brain more susceptible to amyloid accumulation and progressive neuronal loss.

## 4. δ-Secretase (AEP) and the C/EBPβ Pathological Axis

Asparagine endopeptidase (AEP), a cysteine protease, has been identified as δ-secretase [63]. This enzyme fundamentally operates as an endo/lysosomal protease [64]. Both the expression and enzymatic activity of AEP increase in an age-dependent manner in the brain [65]. AEP is implicated in early pathological processes underlying AD, where it mediates the proteolytic cleavage of both APP and microtubule-associated protein Tau [66]. Thus, AEP activation serves as a mechanistic link between cellular aging and the emergence of amyloid and tau pathologies [67].

The pathological upregulation of δ-secretase is regulated by the CCAAT-enhancer binding protein beta (C/EBPβ) transcription factor, a signalling axis influenced by the loss of neurotrophic support and external stressors [26]. Loss of BDNF/TrkB signalling in neurons increases the release of IL-1β, IL-6, and TNF-α, which activate the JAK2/STAT3 pathway. Activated STAT3 binds to the C/EBPβ promoter, inducing its transcriptional upregulation and initiating the C/EBPβ–δ-secretase–APP/tau pathogenic cascade in AD models [25]. Environmental risk factors such as traumatic brain injury (TBI), high-fat diet (HFD), and chronic cerebral hypoperfusion (CCH) act as upstream triggers that independently activate C/EBPβ in the brain [68,69,70]. In BDNF or TrkB hemizygous mice, this activation is further increased, indicating a synergistic interaction between environmental insults and reduced neurotrophic signalling. The resulting transcriptional upregulation of C/EBPβ promotes expression and activation of its downstream effector δ-secretase (AEP), thereby facilitating APP and tau cleavage and contributing to AD-like neurodegeneration [25,65].

Upon activation by the C/EBPβ axis, AEP executes a dual proteolytic mechanism targeting both APP and tau [71]. Specifically, δ-secretase cleaves the ectodomain of APP at distinct asparagine residues (N373 and N585) [27], inducing structural alterations that facilitate subsequent Aβ production by reducing steric constraints for β-site APP-cleaving enzyme 1 (BACE1) [71,72,73]. Concurrently, AEP cleaves tau at asparagine residues N255 and N368 [74]. This structural truncation abolishes the native microtubule-assembly function of tau [64]. In addition, the resulting tau 1–368 fragment is highly neurotoxic and promotes aggregation processes that contribute to neurofibrillary tangle formation [8,75].

Under chronic neuroinflammation, C/EBPβ and AEP form a self-reinforcing loop that amplifies amyloid and tau pathology, driven in part by IL-1, IL-6, and TNF-α–mediated glial activation [63,76]. Inflammatory activation of AEP (δ-secretase) generates Aβ peptides and APP fragments (C586–695), which further enhance C/EBPβ transcriptional activity and sustain neuroinflammatory signalling. This C/EBPβ–δ-secretase feedback loop upregulates C/EBPβ and AEP, driving continued APP and tau cleavage, increased Aβ production, and tau hyperphosphorylation [24,25,27]. Collectively, this self-propagating inflammatory cycle contributes to the spatiotemporal spread of amyloid plaques and tau pathology, ultimately leading to synaptic degeneration and cognitive impairment [77].

## 5. γ-Secretase Modulation

Unlike β-secretase and δ-secretase, γ-secretase is particularly relevant in AD not only because of its constitutive role in APP cleavage, but also because its catalytic activity can be selectively tuned by inflammation-responsive modulatory proteins. The γ-secretase intramembrane protease consists of four essential core subunits-presenilin, nicastrin, anterior pharynx-defective-1 (APH-1), and presenilin enhancer-2 (PEN-2)-which assemble in a defined stoichiometry to form the active catalytic complex [78,79]. However, despite the ubiquitous expression and assembly of these components, a substantial fraction of γ-secretase complexes remains catalytically inactive or in a low-activity state [80]. To adapt to changes in the cellular environment, γ-secretase can associate with γ-secretase modulatory proteins (GSMPs), non-essential transient interactors that fine-tune complex activity and substrate specificity by shifting the protease between low- and high-activity states [80,81,82]. In the context of AD, this modulatory plasticity is especially important because it provides a mechanistic framework through which neuroinflammatory signals may bias γ-secretase activity toward amyloidogenic APP processing. Beyond accessory protein interactions, emerging evidence suggests that inflammatory cues can also affect the functional state and stability of the core γ-secretase complex itself, which comprises four essential integral membrane subunits: presenilin (PSEN1 or PSEN2, harboring the catalytic aspartyl dyad), nicastrin, APH-1, and PEN-2 [22]. Inflammatory mediators such as TNF-α activate c-Jun N-terminal kinase (JNK), which directly phosphorylates presenilin-1 and nicastrin at specific serine/threonine residues, enhancing the stability of the PS1 C-terminal fragment and stimulating γ-secretase activity, a pivotal step in the assembly of functional complexes [83]. TNF-α also promotes expression of PEN2 and nicastrin in neurons, while γ-secretase–dependent Aβ production feeds back to induce TNF-α in glia, establishing a bidirectional cytokine–γ-secretase regulatory loop [84].

A particularly relevant example of this context-dependent modulation is interferon-induced transmembrane protein 3 (IFITM3), an innate immunity factor that directly links neuroinflammatory signalling to amyloidogenesis. During neuroinflammation, cytokines released by activated microglia and astrocytes upregulate IFITM3 expression in neurons and glial cells, where IFITM3 associates with γ-secretase and enhances APP processing and amyloid-β production [22,23,85]. Mechanistically, newly synthesized IFITM3 interacts with the presenilin-1 N-terminal fragment at or near an allosteric regulatory site, thereby selectively increasing γ-secretase activity toward APP C-terminal fragments and promoting the production of Aβ40 and Aβ42 [22,23,85]. Although this response may retain a physiological role within antimicrobial innate immunity, its chronic activation in the AD brain is likely to become maladaptive, favouring persistent amyloid accumulation and disease progression [22,23,85,86].

A second, though more controversial, example of amyloidogenic γ-secretase modulation is γ-secretase activating protein (GSAP). The 16 kDa C-terminal fragment of GSAP has been reported to associate with presenilin-1-containing γ-secretase and the APP C-terminal fragment, forming a ternary complex that selectively promotes APP-C99 cleavage and increases Aβ40/42 production, particularly Aβ42, while largely sparing Notch processing [87,88]. However, the magnitude and specificity of this effect remain debated, as several studies suggest that GSAP may also influence APP trafficking and substrate availability rather than acting exclusively as a dedicated γ-secretase activator [89]. For this reason, GSAP should be interpreted less as definitive proof of a single mechanism and more as additional evidence that γ-secretase output can be shaped by accessory modulators that alter APP-directed processing [82,87,89].

The translational importance of these findings lies in the possibility of modulating γ-secretase activity without reproducing the toxicity associated with broad enzymatic inhibition. Classical γ-secretase inhibitors bind presenilin at or near the active or substrate-binding site and indiscriminately suppress the processing of multiple substrates, including Notch-1, thereby causing the serious on-target adverse effects observed in clinical trials [90,91]. The gastrointestinal, immune-related, and cognitive toxicities associated with pan-Notch inhibition underscore the need for Notch-sparing therapeutic strategies in AD [92,93]. In this context, endogenous γ-secretase modulatory proteins such as IFITM3 and GSAP, together with small-molecule γ-secretase modulators, support the biological feasibility of selectively reducing aggregation-prone Aβ species while largely preserving Notch and other physiologically required γ-secretase-dependent pathways [92]. The therapeutic relevance of γ-secretase in this review does not rest on the enzyme as a static proteolytic complex, but on its capacity to act as an inflammation-sensitive and potentially druggable platform for selective modulation of amyloidogenic processing.

These converging mechanisms are summarized in Figure 2, which integrates transcriptional regulation (STAT3/NF-κB/C/EBPβ), γ-secretase modulation, and post-transcriptional miRNA-mediated control of secretase imbalance in AD.

## 6. The Glial-Secretase Feedback Loop and Synaptic Collapse

The regulation of astrocytic intracellular calcium (Ca^2+^) signalling represents a critical node in the pathogenesis of AD, as exposure to Aβ oligomers induces severe Ca^2+^ dysregulation. In astrocytes, cell surface receptors, including purinergic receptors, metabotropic glutamate receptor 5, and N-methyl-D-aspartate receptors, strictly govern this process by interacting with the inositol trisphosphate receptor (IP3R) located on the endoplasmic reticulum [10,94]. Aβ42 activates these receptors, promoting the release of Ca^2+^ from the endoplasmic reticulum directly into the cytoplasm. The administration of nobiletin, a polymethoxylated flavonoid [95], attenuates this Aβ42-induced increase in intracellular Ca^2+^ levels [10,96]. Mechanistically, nobiletin modulates IP3R, reducing its protein expression in the presence of Aβ42. Furthermore, molecular docking analysis suggests a direct interaction between nobiletin and IP3R, supporting its role in limiting aberrant endoplasmic reticulum calcium release and astrocytic dysfunction [10].

The accumulation of Aβ promotes the assembly of the NOD-, LRR- and pyrin domain-containing protein 3 (NLRP3) inflammasome in glial cells, initiating a feedback loop that exacerbates amyloidogenesis through BACE1 upregulation [97,98,99]. Once activated, the NLRP3 inflammasome recruits the adaptor protein ASC and pro-caspase-1. This complex catalyzes the proteolytic cleavage of pro-caspase-1 into its active form, which subsequently drives the maturation and secretion of the pro-inflammatory cytokine interleukin-1β [100]. Glia-derived interleukin-1β stimulates adjacent neurons to upregulate BACE1 expression, thereby amplifying the amyloidogenic proteolytic processing of the APP [101]. Pharmacological intervention utilizing *Picrorhiza kurroa*, a medicinal herb recognized for its anti-inflammatory properties [102], effectively suppresses the activation of the NLRP3 inflammasome, leading to a marked decrease in interleukin-1β secretion [103]. *Picrorhiza kurroa* downregulates BACE1 expression [103], by inducing the SIRT1–PPAR-γ signalling pathway [104]. Similarly, the compound Dl-3-n-Butylphthalide abrogates this neuroinflammatory cascade by inhibiting the physical interaction between thioredoxin-interacting protein (TXNIP) and NLRP3 [105]. This induces the suppression of caspase-1 activation and reduces interleukin-1β release [105,106,107], thereby repressing the pathological upregulation of BACE1. Ultimately, these interventions dismantle the inflammation-driven amyloidogenic feedback loop [105,107].

Within the inflammatory milieu of the Alzheimer’s brain, the precise phenotypic polarization of microglia dictates the efficiency of Aβ clearance and the trajectory of neurodegeneration [108]. Chronic exposure to Aβ peptides triggers a classically activated M1-like state [109,110]. The pro-inflammatory M1 microglial phenotype contributes directly to neurotoxicity, oxidative stress, and synaptic damage through the sustained release of mediators such as nitric oxide and reactive oxygen species [111]. M1-polarized microglia constitutively secrete high levels of pro-inflammatory cytokines, including interleukin-1β (IL-1β), tumor necrosis factor-alpha (TNF-α), and interleukin-6 (IL-6), which disrupt synaptic structure and function and promote neuronal loss [111,112]. In AD, chronic exposure to amyloid-β (Aβ) drives microglia toward a persistent pro-inflammatory state, where M1-like phenotypes increasingly predominate over reparative M2-like profiles as pathology advances [112]. Under these conditions, microglia become highly neurotoxic and exhibit phagocytic exhaustion, with impaired autophagy and reduced capacity to clear Aβ aggregates [112,113]. The M2 microglial phenotype is an anti-inflammatory, neuroprotective state that releases cytokines such as interleukin-10 (IL-10), interleukin-4 (IL-4), and transforming growth factor-β (TGF-β) to restrain neuroinflammation and support tissue repair [108,114]. Therapeutic strategies for neurodegenerative diseases increasingly emphasize modulation of microglial polarization, promoting a shift from pro-inflammatory M1 states toward protective M2-like phenotypes [115]. Inducing the transition from an M1 to an M2 phenotype has been shown to restore microglial homeostasis, enhance Aβ uptake and phagocytic activity, and promote the enzymatic and lysosomal degradation of pathological protein aggregates [108].

Unchecked pathological crosstalk between glial cells and synaptic signalling pathways drives profound remodeling and degeneration of tripartite synapses, characterized by loss of astrocytic coverage, impaired neurotransmitter clearance, and structural collapse of pre- and postsynaptic elements [116]. Under physiological conditions, astrocytes ensheathe synapses with peri-synaptic processes, forming the tripartite synapse and providing structural support that stabilizes presynaptic terminals and postsynaptic spines [117]. In this healthy state, astrocytes secrete synaptogenic and matricellular molecules and organize the peri-synaptic extracellular matrix, which are required for synapse formation, maturation, and long-term maintenance of precise pre- and postsynaptic communication [118].

Aβ accumulation in the perisynaptic space destabilizes astrocytic Ca^2+^ homeostasis, leading to aberrant, state-dependent Ca^2+^ signals that alter the timing and magnitude of gliotransmitter release [119]. Modelling and experimental work show that Aβ-related changes in mGluRs and PMCAs drive Ca^2+^ overload and desynchronize Ca^2+^ transients from glutamate/ATP release, depleting vesicle pools and compromising precise gliotransmission [120,121]. This contributes to glutamate dysregulation and excitotoxic synapse loss [121]. Aβ and tau shift microglia from protective to neurotoxic, pro-inflammatory states, with sustained cytokine release (IL-1β, TNF-α, IFN-γ) and complement-mediated synaptic engulfment that correlates with synapse loss and cognitive decline [122,123]. Astrocytes and microglia mutually amplify activation via cytokines, chemokines, and NF-κB–dependent pathways, creating a positive feedback loop of neuroinflammation and structural degradation [123]. Synaptic loss is considered an early event in AD and represents the strongest correlate of cognitive impairment [124], with converging evidence from animal models and in vivo imaging supporting its occurrence prior to overt neuronal death [125]. Reactive astrocytes around Aβ plaques lose homeostatic support, while activated microglia excessively prune or engulf synapses and dystrophic neurites, collapsing tripartite synaptic structure and function. This provides a mechanistic link by which upstream Aβ/secretase dysregulation is converted into widespread synaptic failure that defines the clinical syndrome [126].

## 7. Neuroinflammatory Mechanisms of Synaptic Disintegration and Proteostatic Failure

The neuroinflammatory milieu characteristic of AD contributes to structural alterations of the postsynaptic density, impairing synaptic architecture and transmission [41]. Chronic neuroinflammation, including that induced by metabolic stressors such as high-fat diet, is associated with a marked downregulation of the key scaffolding protein PSD-95 in the hippocampus [39,127]. Concurrently, the receptor for activated C kinase 1 (RACK1), a WD40 repeat-containing scaffolding protein, shows reduced membrane-associated expression during disease progression [128]. Under physiological conditions, RACK1 interacts with the NR2B subunit of N-methyl-D-aspartate (NMDA) receptors, limiting its phosphorylation by the Fyn kinase and thereby restraining excessive calcium influx [128]. Inflammatory conditions leading to RACK1 depletion may therefore disrupt NMDA receptor regulation, affecting receptor gating and trafficking and contributing to synaptic dysfunction. In addition, RACK1 interacts with the intracellular domains of the β1 and β3 subunits of gamma-aminobutyric acid A (GABA _A_) receptors, where it contributes to the modulation of inhibitory neurotransmission [128].

To maintain synaptic structural integrity, continuous clearance of misfolded proteins and the local synthesis of structural components are required, both of which are impaired during AD pathogenesis. Structural plasticity of dendritic spines depends on the local translation of β-actin mRNA within the growth cone, a process regulated by RACK1 through its interaction with the β-actin mRNA/Zipcode-binding protein 1 (ZBP1) complex at the ribosome [75]. In parallel, the ubiquitin-proteasome system (UPS) contributes to proteostasis by targeting aberrant proteins for degradation via specific E3 ubiquitin ligases, including carboxyl terminus of HSC70 interacting protein (CHIP) and ubiquitin carboxyl-terminal esterase-1 (UCHL-1), both of which are reduced in the AD brain. Therapeutic interventions such as forced treadmill exercise actively upregulate the expression of these E3 ligases, fundamentally restoring proteostasis [129]. Mechanistically, increased CHIP levels promote its interaction with heat shock protein 70 (HSP70), facilitating the recognition and ubiquitination of hyperphosphorylated Tau, for proteasomal degradation. In addition, upregulation of UCHL-1 protein has been associated with enhanced degradation of BACE1, thereby reducing amyloidogenic APP processing and Aβ production [129,130,131].

The intrinsic vulnerability of the hippocampal network to neuroinflammatory and proteostatic collapse is largely determined by its exceptionally high bioenergetic demands [132,133]. Hippocampal neurons, along with those in selected cortical regions, show elevated rates of ATP production and oxygen consumption, rendering them particularly sensitive to reactive oxygen species [134,135]. This metabolic vulnerability is further exacerbated by the accumulation of Aβ, which preferentially affects brain regions characterized by high neuronal activity and energy demand [132,133,136]. Exposure to Aβ assemblies in these metabolically active regions rapidly induces oxidative stress, which in turn promotes the activation of pro-inflammatory mediators [136]. The resulting localized neuroinflammation triggers microglial activation and the release of cytokines such as IL-1β and TNF-α, which have been shown to upregulate BACE1 expression in neighboring neurons [137,138]. This establishes a self-amplifying loop in the hippocampus, where elevated metabolic stress promotes inflammatory signalling that further enhances amyloidogenic processing and synaptic dysfunction.

Within this pathogenic cascade, PI3K–Akt signalling plays a critical role in maintaining synaptic integrity and limiting neuronal loss [139,140]. In AD-like conditions, Aβ oligomers suppress PI3K/Akt signalling, leading to activation of Forkhead box O (FoxO) transcription factors, induction of pro-apoptotic molecules such as Puma, and increased neuronal apoptosis [141]. In contrast, pharmacological or receptor-mediated enhancement of PI3K/Akt signalling promotes Akt-dependent phosphorylation of glycogen synthase kinase-3β (GSK-3β) at Ser9, thereby inhibiting this pivotal Tau kinase and reducing Tau hyperphosphorylation [141,142].

PI3K/Akt activation also upregulates heat-shock factor 1 (HSF1) and downstream HSP70, contributing to the maintenance of proteostasis and facilitating the clearance of misfolded proteins through pathways including the UPS [141,143]. In experimental models, it has been shown that interventions that enhance PI3K/Akt signalling, such as regular physical exercise and selected natural compounds, increase hippocampal PI3K catalytic subunits and Akt phosphorylation at Ser473, while attenuating GSK-3β activity [144]. Sustained activation of PI3K/Akt signalling may therefore counteract multiple neurotoxic kinase pathways, while also reducing BACE1 expression and Aβ-induced apoptotic signalling, providing a broad neuroprotective mechanism that preserves synaptic function and slows cognitive decline [145].

## 8. Multi-Target Therapeutic Strategies Targeting the Neuroinflammation–Secretase Axis

Recent pharmacological approaches employing bioactive natural compounds have shown promising efficacy in attenuating oxidative stress–induced damage and dysregulated secretase activity in AD models [146,147]. Nobiletin, a polymethoxylated citrus flavonoid, has shown promising effects to ameliorate STZ-induced AD-like pathology through activation of SIRT1/FoxO3a-dependent autophagy. These effects are associated with reduced BACE1 activity, decreased hippocampal Aβ levels, and attenuation of oxidative stress and neuroinflammatory responses [41].

Ginsenoside Rg1 has been shown to exert robust neuroprotective effects by modulating both amyloidogenic processing and Tau pathology. In experimental AD models, it is associated with reduced BACE1 expression and Aβ_1–42_ deposition, decreased Tau phosphorylation, increased antioxidant defenses, and modulation of apoptotic and neuroinflammatory pathways, potentially through normalization of the Wnt/GSK-3β/β-catenin signalling cascade [148,149].

The marine xanthophyll astaxanthin has been shown to exert protective effects against LPS-induced neuroinflammation, oxidative stress, and memory impairment. Mechanistically, it has been reported to interact with the DNA-binding and linker domains of STAT3, thereby inhibiting its transcriptional activity. This is associated with reduced β-secretase expression and decreased Aβ_1–42_ production in both in vitro and in vivo models [150]. More broadly, astaxanthin downregulates STAT3-dependent inflammatory signalling and amyloidogenesis and has been proposed as a multi-target neuroprotective agent in AD models [150,151,152].

**Figure 3:**
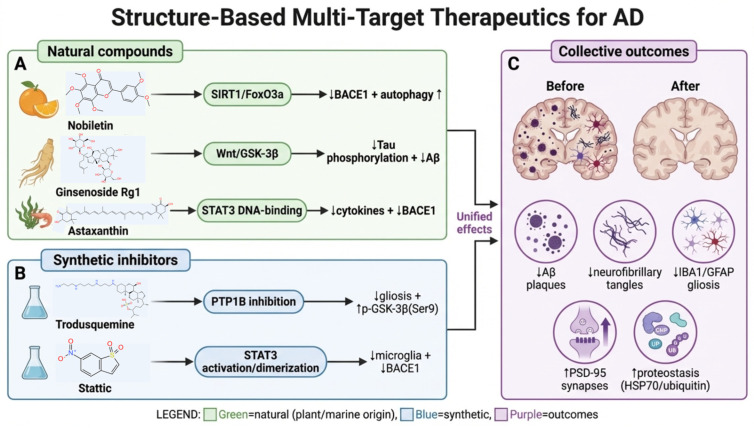
Potential outcomes of multi-target therapeutics interrupting the neuroinflammation–secretase axis. (**A**) Natural compounds [nobiletin (citrus flavonoid, CID 72344), ginsenoside Rg1 (ginseng saponin, CID 441923), astaxanthin (marine xanthophyll, CID 5281224)]. (**B**) Synthetic inhibitors [trodusquemine (cholesterol-spermine conjugate, CID 9917968), Stattic (nitrothiophene derivative, CID 2779853)]. (**C**) Collective downstream effects reducing Aβ/Tau pathology, gliosis, and synaptic loss while restoring proteostasis. Chemical structures stylized from chemical classes; CIDs from PubChem.

Beyond naturally derived molecules, the use of highly specific synthetic inhibitors represents a complementary strategy to modulate neuroinflammatory pathways in AD. Pharmacological inhibition of protein tyrosine phosphatase 1B (PTP1B) using trodusquemine in APP-based AD mouse models has been shown to prevent hippocampal neuron loss, improve spatial memory and attenuates neuroinflammatory responses [153]. Systemic administration of trodusquemine reduces IBA1/GFAP-defined gliosis and restores inhibitory phosphorylation of GSK-3β, a key Tau kinase [153,154]. In a BACE1-overexpressing co-morbidity model (PLB4), trodusquemine decreases APP levels, attenuates astrogliosis, normalizes expression of the ER chaperone BiP/GRP78, and improves glucose tolerance, linking PTP1B inhibition to both metabolic and neuroinflammatory improvements [155]. Consistent with these findings, reviews have highlighted PTP1B inhibition as a multitarget strategy capable of modulating ER-stress signalling, microglial activation, and synaptic dysfunction in AD and related disorders [154]. In a parallel immunomodulatory axis, systemic inflammation or Aβ exposure increases phosphorylated STAT3 in the hippocampus, promoting microglial and astrocyte activation, cytokine release (IL-1β, IL-6, TNF-α), and upregulation of BACE1, thereby enhancing Aβ production [11,13,156]. The small-molecule Stattic, which inhibits STAT3 activation, dimerization, and nuclear translocation, reduces LPS-induced microglial activation, lowers brain cytokines, and significantly decreases hippocampal BACE1 levels in vivo [11]. In Aβ-based mouse models, STAT3 inhibition (Stattic or other STAT3-specific drugs) attenuates reactive astrogliosis, reduces Aβ plaque load and cerebral amyloid angiopathy, normalizes network activity, and improves learning and memory [47,157]. These therapeutic approaches are summarized in Figure 3.

From a rational drug design perspective, these compounds share a common mechanistic rationale: by simultaneously targeting multiple nodes of the neuroinflammation–secretase feedback loop, they circumvent the limitations of single-target strategies [158]. The underlying principle of multitarget design in AD rests on the observation that key pathological pathways—NF-κB/STAT3-mediated transcription of BACE1, C/EBPβ-driven δ-secretase upregulation, miRNA-mediated suppression of ADAM10, and glial NLRP3 inflammasome activation—are not independent but form an interconnected network in which perturbation of a single node is rapidly compensated by others [159]. Compounds such as nobiletin, ginsenoside Rg1, and astaxanthin share structural and physicochemical features (lipophilicity, blood–brain barrier permeability, pleiotropic anti-inflammatory scaffolds) that enable them to engage several of these nodes simultaneously [160]. A systematic approach to designing such inhibitors involves: (1) network pharmacology analysis to identify hub proteins at the intersection of neuroinflammatory and amyloidogenic pathways [161]; (2) computational docking and ADMET prediction to prioritize scaffolds with polypharmacological activity [162]; and (3) fragment-based or bioisosteric optimization to tune selectivity and minimize off-target toxicity [163].

## 9. Materials and Methods

A comprehensive literature search was conducted across the PubMed database, covering research published between January 2016 and March 2026. The primary search strategy utilized the Boolean operators: “Neuroinflammation AND Secretase Regulation in Alzheimer’s Disease”. To ensure the inclusion of contextually relevant studies and high-impact mechanistic data, the AI-powered search engine Consensus was employed to identify and prioritize peer-reviewed articles matching the specific research framework and sub-topics of this review. All scientific illustrations and schematic representations (Figure 1, Figure 2 and Figure 3) were made using BioRender.com (Created in https://BioRender.com, accessed on 4 May 2026).

## 10. Conclusions

Collectively, the literature data highlights a major role for neuroinflammation and secretase dysregulation in AD. These processes constitute a closely interconnected pathogenic network that contributes to both amyloid and Tau pathology. This perspective underscores the importance of therapeutic strategies aimed at interrupting this self-propagating cycle. Building on this mechanistic framework, current and emerging interventions should be evaluated not only for their ability to reduce Aβ burden, but also for their ability to modulate glial phenotypes, restore non-amyloidogenic APP processing, and support proteostatic and metabolic resilience in vulnerable hippocampal circuits. In this context, multi-target strategies integrating bioactive natural compounds, selective secretase modulators, and inhibitors of key inflammatory hubs (including STAT3, PTP1B, or NLRP3) may offer a promising avenue for disease modification, although their translational efficacy and safety profiles remain to be fully established.

A particularly promising direction for future research lies in defining how epigenetic regulators, including both miRNAs and selected lncRNAs, interact with inflammatory signalling to modulate secretase activity and amplify amyloidogenic APP processing in AD. In this perspective, non-coding RNA networks may represent not only mechanistic amplifiers of disease progression but also a potential source of more selective therapeutic targets within the neuroinflammation–secretase axis.

The complexity of this bidirectional feedback loop suggests that classical single-target therapeutic paradigms are fundamentally insufficient to halt the irreversible progression of AD. Historical approaches based on single-modality drugs, such as broad-spectrum γ-secretase inhibitors, failed decisively in clinical trials due to their indiscriminate attenuation of essential physiological pathways. Complete inhibition of γ-secretase disrupts the essential proteolytic processing of the Notch-1 receptor, which has been associated with severe side effects including gastrointestinal bleeding and a paradoxical worsening of cognition. These findings highlight the limitations of a “one-molecule one-target” strategy to adequately address the multifactorial nature of AD, encompassing oxidative stress, neuroinflammation, and amyloid toxicity. Strategies that selectively modulate the inflammatory context, such as targeting transiently binding γ-secretase modulatory proteins or leveraging pleiotropic natural compounds, may help attenuate the amyloidogenic pathway while minimizing systemic toxicity. Ultimately, modulation of the neuroinflammatory milieu represents a promising therapeutic direction, potentially acting upstream of secretase dysregulation and contributing to the preservation of synaptic integrity in the context of progressive inflammatory stress. In summary, therapeutic strategies that simultaneously target neuroinflammatory signalling and secretase activity may be required to translate mechanistic insights into clinically meaningful, long-term benefits in AD.

## Data Availability

No new data were created or analyzed in this study. Data sharing is not applicable to this article.

## Abbreviations

The following abbreviations are used in this manuscript:

AD	Alzheimer’s disease
Aβ	Amyloid-beta
AEP	Asparagine endopeptidase
ADAM10	A disintegrin and metalloproteinase domain-containing protein 10
APP	Amyloid precursor protein
APP-CTF	Amyloid precursor protein C-terminal fragment
BACE1	Beta-site amyloid precursor protein-cleaving enzyme 1
BDNF	Brain-derived neurotrophic factor
BBB	Blood–brain barrier
C/EBPβ	CCAAT/enhancer-binding protein beta
CCH	Chronic cerebral hypoperfusion
CHIP	Carboxyl terminus of HSC70-interacting protein
ER	Endoplasmic reticulum
FoxO	Forkhead box O
GABA	Gamma-aminobutyric acid
GFAP	Glial fibrillary acidic protein
GSAP	Gamma-secretase activating protein
GSMPs	Gamma-secretase modulatory proteins
GSK-3β	Glycogen synthase kinase-3 beta
HFD	High-fat diet
HIF-1α	Hypoxia-inducible factor-1 alpha
HRE	Hypoxia response element
HSF1	Heat-shock factor 1
HSP70	Heat shock protein 70
IBA1	Ionized calcium-binding adaptor molecule 1
IDE	Insulin-degrading enzyme
IFITM3	Interferon-induced transmembrane protein 3
IKK	IκB kinase
IL	Interleukin
IP3R	Inositol trisphosphate receptor
JAK2	Janus kinase 2
lncRNA	Long non-coding RNA
LPS	Lipopolysaccharide
mGluR5	Metabotropic glutamate receptor 5
miRNA	MicroRNA
MyD88	Myeloid differentiation primary response 88
NF-κB	Nuclear factor kappa-light-chain-enhancer of activated B cells
NLRP3	NOD-, LRR- and pyrin domain-containing protein 3
NMDA	N-methyl-D-aspartate
PEN-2	Presenilin enhancer-2
PI3K	Phosphoinositide 3-kinase
PKR	Protein kinase R
PSD-95	Postsynaptic density protein 95
PTP1B	Protein tyrosine phosphatase 1B
RACK1	Receptor for activated C kinase 1
RAGE	Receptor for advanced glycation end products
ROS	Reactive oxygen species
sAPPα	Soluble amyloid precursor protein alpha
SIRT3	Sirtuin 3
SORL1	Sortilin-related receptor 1
STAT3	Signal transducer and activator of transcription 3
STZ	Streptozotocin
TBI	Traumatic brain injury
TGF-β	Transforming growth factor beta
TLR	Toll-like receptor
TNF-α	Tumor necrosis factor alpha
TrkB	Tropomyosin receptor kinase B
TXNIP	Thioredoxin-interacting protein
UCHL-1	Ubiquitin carboxyl-terminal hydrolase L1
UPS	Ubiquitin–proteasome system
UTR	Untranslated region
ZBP1	Zipcode-binding protein 1
